# Liver Transplantation for Fulminant Hepatic Failure Precipitated by Pheochromocytoma Crisis in the Setting of Using Garcinia cambogia Weight Loss Supplement: A Case Report

**DOI:** 10.7759/cureus.36045

**Published:** 2023-03-12

**Authors:** Motaz A Selim, Krystal Weierstahl, Calvin Eriksen, Terra Pearson, Harvey Woehlck

**Affiliations:** 1 Division of Transplant Surgery, Department of Surgery, Medical College of Wisconsin, Milwaukee, USA; 2 Department of Anesthesiology, Medical College of Wisconsin, Milwaukee, USA

**Keywords:** acute liver failure, weight loss supplement, complications, garcinia cambogia, weight loss supplements complications, pheochromocytoma multisystem crisis, acute liver failure (alf), orthotopic liver transplantation

## Abstract

We report the first case of liver transplantation for fulminant hepatic failure precipitated by hepatocellular injury due to the synergistic effect of pheochromocytoma crisis and simultaneous use of *Garcinia cambogia*. Complex diagnosis and treatment decisions are discussed, as well as possible pathophysiology that led to liver failure.

## Introduction

Pheochromocytoma crisis (PCC) is a rare potentially fatal pheochromocytoma presentation [1]. PCC presents acutely with hemodynamic instability and end-organ damage. There are no published cases of liver transplantation (LT) for fulminant hepatic failure (FHF) secondary to PCC. We are reporting the first such case and describing the diagnostic and management challenges caused by the conflicting effect exerted by FHF and PCC on hemodynamics in a critically ill patient. The patient was also using a weight loss supplement containing *Garcinia cambogia*, which has previously been reported to cause acute liver injury that may progress to FHF requiring LT [2-5].

## Case presentation

A 47-year-old Caucasian female with no relevant past medical history presented with palpitations, nausea, and vomiting. She deteriorated rapidly in the emergency room with altered mental status and circulatory collapse requiring intubation and initiation of vasopressors. Her workup was significant for elevated white blood cell count (24 x 10^3^/ml^3^), creatinine (2.13 mg/dL), and liver chemistries, including alanine transaminase (ALT) > 7,000 unit/L, international normalized ratio (INR) > 8, alkaline phosphatase at 173 unit/L, total bilirubin at 1.2 mg/dL, and direct bilirubin at 0.8 mg/dL, denoting a pattern of hepatocellular injury. Her previous blood chemistries were reported to be normal on previous routine checks with her primary care physician. She was subsequently transferred to our transplant intensive care unit (TICU).

General examination was significant for mottling of extremities with palpable peripheral pulses. Glasgow Coma Scale (GCS) score remained 7-9 off sedation. She was hypotensive requiring norepinephrine infusion to maintain mean arterial pressure of 65 mmHg. Continuous renal replacement therapy (CRRT) was initiated for acute kidney injury. Lactic acid was 8.5 mmol/L. Liver chemistries on post-admission day three showed aspartate aminotransferase (AST) at 12,000 unit/L, ALT at 5,000 unit/L, lactate dehydrogenase at 13,000 unit/L, alkaline phosphatase at 328 unit/L, and total bilirubin peaked at 2.4 mg/dL (direct: 1.8 mg/dL). Factors V and VII activities were 18% and 22%, respectively.

Critical care management was conducted per TICU guidelines. Blood and respiratory cultures were negative. N-acetylcysteine was started for non-acetaminophen FHF. No clear etiology for FHF was identified at this point. Even though the patient had started taking an over-the-counter weight loss supplement containing *Garcinia cambogia*, a compound known for its potential hepatotoxicity [2-5], for the past three days, drug-induced liver injury (DILI) etiology was deemed unlikely based on Roussel Uclaf Causality Assessment Method (RUCAM) [6] score of 2. The workup for potential causes of FHF and possible chronic liver disease was negative and included testing for hepatotropic and non-hepatotropic viral hepatitis and autoimmune and metabolic liver disease markers. Alcohol levels were negative on admission and there was no significant alcohol use history per family. Liver ultrasonography with vascular duplex was negative for any abnormality. A liver biopsy was obtained and showed 50% zone 3 necrosis with no significant fibrosis, inflammation, or steatosis in the viable liver. The biopsy was consistent with ischemic liver injury but could not exclude possible toxins or DILI as well. Expedited evaluation for LT was initiated based on King's College Criteria for non-acetaminophen FHF and worsening clinical status. The patient was listed for LT as a status 1A.

Given signs of cerebral edema on computed tomography of the head, a subarachnoid bolt device was placed to monitor the intracranial pressure (ICP). ICP was maintained at <20 mmHg with the head of the bed elevation. Lying flat caused ICP elevation with systemic hypertension (systolic pressure: 200 mmHg) initially thought to be due to the Cushing reflex, requiring temporary switching from norepinephrine infusion to nicardipine infusion. The bolt was exchanged to an external ventricular drain (EVD) to allow drainage of cerebrospinal fluid (CSF) for the management of the ICP.

**Figure 1:**
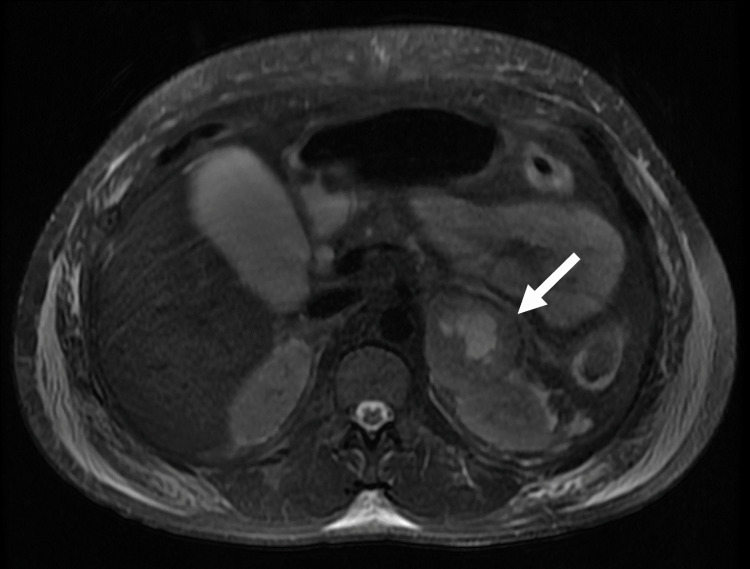
MRI T2-weighted image. Arrow pointing at the left adrenal lesion.

Abdominal magnetic resonance imaging (MRI) was obtained for transplant evaluation. A partially necrotic left adrenal mass (5 x 5 cm) was incidentally noted (Figure 1).

There was no radiological evidence of malignancy. We planned to continue management with norepinephrine for hypotension and nicardipine for hypertension and wait to perform left adrenalectomy after LT. Results of plasma metanephrine and normetanephrine levels were received post-transplant. Despite the norepinephrine infusion and CRRT, both known to be associated with false-positive results [7], the results were markedly elevated confirming the diagnosis of pheochromocytoma (>50 and 24 nmol/L, upper limits of 0.49 and 0.89, respectively).

**Figure 2:**
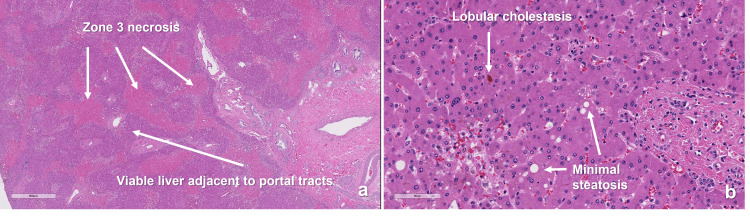
Liver explant pathology slides (hematoxylin and eosin stain). (a) 2x magnification showing extensive zone 3 necrosis (~60%). (b) 20x magnification showing bland lobular cholestasis with minimal steatosis and no significant fibrosis or inflammation.

The patient received LT using standard criteria brain dead donor liver three days after listing. A standard cava replacement approach was performed with the aid of total mesenteric and peripheral venovenous bypass (for hemodynamic lability) and intraoperative CRRT [8]. Reperfusion was uneventful. Due to the patient's instability, deferring the adrenalectomy until postoperative day (POD) one was felt to be safer to allow for the improvement of liver functions and patient hemodynamics. By the same token, a staged biliary reconstruction during the planned return to the operating room the next day was felt to be safer and consistent with the center's previously published experience [9]. The surgical procedures were uncomplicated. Explanted liver pathology showed findings similar to the pre-transplant biopsy (Figure 2).

**Figure 3:**
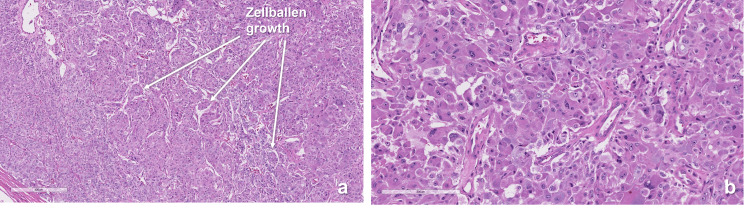
Pheochromocytoma pathology slides (hematoxylin and eosin stain). (a) 10x magnification showing encapsulated growth with sheets of cells and a zellballen pattern. (b) 20x magnification showing oncocytic cells with abundant eosinophilic and granular cytoplasm.

The adrenal mass showed oncocytic pheochromocytoma with no atypical mitoses or capsular or vascular invasion (Figure 3).

Intraoperative management was remarkable for a hypertensive baseline despite the adequate depth of anesthesia instead of the vasodilated high cardiac output state expected in hepatic failure patients. Episodes of systemic pressures up to 280/160 with minimal response to phentolamine occurred frequently with ICP elevation to 45 mmHg. Withdrawing 10-15 mL of CSF resulted in a prompt reduction of ICP and simultaneous resolution of the hypertensive crisis.

Mental status improved after LT. Liver chemistries normalized promptly. Norepinephrine was stopped on POD three with no hemodynamic lability, and EVD was removed on POD seven. There were no signs of adrenal insufficiency. Oral prednisone 5 mg daily was used as part of the immunosuppression regimen. The kidney functions were never regained post-LT and the patient remains dependent on dialysis until the writing of this manuscript. Mottling of the extremities that was present since initial presentation (most likely related to the PCC, worsened by the intermittent use of vasopressors in the peri-transplant period) slowly resolved, leaving only areas of dry gangrene of the tips of the toes of both feet that was eventually debrided. The patient started having hematochezia on POD 25 and upper and lower gastrointestinal endoscopy failed to identify a definitive source of bleeding. Abdominal angiogram revealed patent mesenteric and colonic vessels and active luminal extravasation in the hepatic flexure. Interventional radiology was unsuccessful in controlling the bleeding, so a right hemicolectomy was performed on POD 29. The bleeding was initially thought to be due to ischemic colitis related to the previous episodes of PCC and the use of vasopressors in the peri-transplantation period; however, pathology examination of the excised colon showed mucosal ulceration and crypt apoptosis with no viral inclusions, suspicious of mycophenolate mofetil-induced injury. The patient was subsequently taken off mycophenolate mofetil. She was discharged to inpatient rehabilitation and then home shortly thereafter. She is now four years from LT and adrenalectomy with excellent liver graft function, normal blood pressure, and is physically active.

## Discussion

We presented a case of LT for FHF precipitated by a PCC causing a profound ischemic liver injury that was probably potentiated by the toxic effect of *Garcinia cambogia*. To our knowledge, this is the only case of such nature to be reported. PCC is a rare but serious presentation of pheochromocytoma, and the incidence is 7-18%. PCC presents with refractory hypotension requiring vasopressor or even mechanical support of the circulation [10], multiple organ dysfunction, and even death [1]. The hyperacuity of presentation, hemodynamic lability, and multi-organ failure support the diagnosis of PCC in our patient. Although DILI was deemed unlikely as the cause of FHF based on the RUCAM causality assessment tool (score of 2) [6] yet, based on the published data about the hepatotoxic effects of *Garcinia cambogia* [2-5], we suspect that its use may have potentiated the hepatocyte injury from the ischemic effects of the PCC. The pre-transplant liver biopsy and the explant pathology showed ischemic liver injury but could not clearly dismiss the possibility of toxic or DILI either. DILI precipitated by unregulated over-the-counter dietary, herbal, and weight loss supplements is being reported with an alarmingly increasing frequency [11], yet these supplements still lack federal regulations. *Garcinia cambogia*, in particular, is frequently reported in association with acute liver injury [3] with some cases progressing to FHF requiring LT [2,4,5].

Liver damage and biochemical signs of diabetes have been reported with pheochromocytoma, and improvement resulted from phenoxybenzamine and surgical resection of the pheochromocytoma [12]. Hepatocellular injury during PCC is related to the abnormal increase in hepatic metabolic rate due to alpha-1 receptor overstimulation by the excess catecholamines produced by the tumor leading to increased cytosolic calcium [13], which in turn stimulates energy-dependent gluconeogenesis and reactive oxygen species production [14]. The use of *Garcinia cambogia* could have amplified the hepatocellular injury caused by the PCC stress through depleting energy substrates leading to relative ischemia. *Garcinia cambogia* contains hydroxycitric acid, which uncouples fatty acid oxidation from the generation of adenosine triphosphate (ATP) [15]. This results in a lack of ATP production from acetyl-CoA by the mitochondria. In addition, hydroxycitric acid reduces the supply of acetyl-CoA by blocking the production of citrate, producing two separate blockages in the supply chain of ATP. Increased energy requirements in the setting of reduced energy availability may lead to a state of relative ischemia, programmed cell death, and cell lysis due to the failure of cellular homeostatic mechanisms [14]. Relative ischemia will also impede hepatocellular regeneration, eventually leading to FHF. The hepatic ischemic injury pattern seen on the explant pathology could have occurred due to relative ischemia as described by the aforementioned mechanism; it can also be caused or augmented by the profound vasoconstriction alternating with hypotensive episodes.

In our case, resection of the pheochromocytoma was felt to be prohibitively high risk in the setting of FHF, brain edema, and hemodynamic fluctuations. The lability precluded the use of alpha-blockade therapy preoperatively. The mainstay of management was to stabilize the patient medically for LT and then perform the adrenalectomy once liver functions are restored. Intraoperative hypertensive crises responded more promptly to lowering ICP than to alpha blockade, probably through a mechanism of reducing sympathetic tone. Many reports demonstrated improved hemodynamics during pheochromocytoma resection with epidural anesthesia using local anesthetics for sympathetic denervation [16,17]. The adrenal tumor pathology showed oncocytic neoplasm positive for chromogranin, synaptophysin, and S100, and negative for inhibin and calretinin, consistent with pheochromocytoma. Adrenocortical oncocytic neoplasms are rare, with only a few cases reported [18]. They are rarely functional, and all reported cases were benign.

## Conclusions

To our knowledge, there are no published cases of LT secondary to PCC. We described management strategies for this unique presentation and peri-transplant challenges faced due to the contradictory hemodynamic effects of FHF and functional pheochromocytoma. Prompt multidisciplinary management in a specialized TICU was key to success, allowing for making complex decisions and implementing them promptly. We also aim to raise awareness of the potential harms associated with rampant use and easy access to unregulated weight loss supplements like *Garcinia cambogia* in the market.
